# Three-Dimensional CBCT Analysis of Second Mesiobuccal Canal Anatomy in Maxillary Molars

**DOI:** 10.3390/diagnostics16091299

**Published:** 2026-04-27

**Authors:** Hanadi Sabban, Maysoon Albahiti, Suha S. Maddah

**Affiliations:** 1 Division of Oral and Maxillofacial Radiology, Department of Oral Diagnostic Sciences, Faculty of Dentistry, King Abdulaziz University, P.O. Box 80209, Jeddah 21589, Saudi Arabia; 2 Department of Endodontics, Faculty of Dentistry, King Abdulaziz University, P.O. Box 80209, Jeddah 21589, Saudi Arabia; mhaji@kau.edu.sa (M.A.); ssmaddah@kau.edu.sa (S.S.M.)

**Keywords:** cone-beam computed tomography (CBCT), mesiobuccal canal, maxillary molars, root canal morphology, endodontic anatomy, three-dimensional imaging, retrospective study

## Abstract

**Background**: This study aimed to evaluate the prevalence, morphology, and influencing factors of the second mesiobuccal (MB2) canal in maxillary molars and the middle-mesial canal in mandibular molars using cone-beam computed tomography (CBCT). **Methods**: A total of 146 CBCT scans acquired between 2023 and 2025 were retrospectively analyzed at the Oral Radiology Division, King Abdulaziz University Dental Hospital. Canal prevalence, morphometric dimensions, symmetry, and Vertucci configurations were recorded and correlated with age, sex, and voxel size. **Results**: MB2 canals were detected in 64.4% of maxillary first molars and 29.6% of second molars, while middle-mesial canals were found in 17.8% of mandibular first molars and 6.4% of second molars. Detection rates declined with increasing voxel size and patient age (*p* < 0.05). Bilateral symmetry was strong for MB2 (κ = 0.78) but moderate for mandibular canals (κ = 0.29). Vertucci Type I and II were most common in maxillary roots, and Type IV predominated in mandibular mesial roots. **Conclusions**: These findings confirm CBCT as a reliable tool for identifying complex canal systems and emphasize the importance of voxel resolution and anatomical understanding in enhancing endodontic diagnosis and treatment success.

## 1. Introduction

The long-term success of endodontic treatment depends on complete cleaning, shaping, and obturation of all root canals within the tooth. Undetected or untreated canals are among the main causes of persistent periapical disease and retreatment. The second mesiobuccal (MB2) canal in maxillary molars is the most frequently missed canal, representing a persistent diagnostic and technical challenge. Detection is complicated by its small diameter, variable trajectory, and tendency to merge apically with the main mesiobuccal canal, which makes identification difficult during clinical and radiographic examination [1,2,3,4,5].

Traditional periapical radiography plays a crucial role in endodontic diagnosis; however, its two-dimensional nature limits visualization of complex canal anatomy and fine internal details such as accessory or secondary canals [6,7]. Consequently, three-dimensional imaging may be indicated when conventional examination and radiography do not adequately explain clinical signs or when complex anatomy is suspected [7,8].

In vitro diagnostic research suggests that adjunctive magnification and CBCT can improve MB2 detection when benchmarked against high-accuracy reference standards such as micro-CT [8]. To strengthen transparency and reproducibility in diagnostic accuracy research in endodontics, dedicated reporting guidance such as the PRIDASE recommendations should be followed [9].

Cone-beam computed tomography (CBCT) provides high-resolution three-dimensional imaging with multiplanar reconstructions, enabling improved visualization of canal morphology with minimal geometric distortion. CBCT allows clinicians to evaluate canal configuration, assess root curvature, and detect anatomical variations that may not be visualized with conventional radiography [10]. Numerous studies have shown that CBCT improves MB2 detection and enhances endodontic treatment planning [10,11,12,13]. Because CBCT involves ionizing radiation, it should be used judiciously with appropriate field-of-view selection and exposure optimization to balance diagnostic benefit against radiation dose and artifacts [13].

Extensive CBCT-based studies across populations have demonstrated substantial variation in MB2 canal prevalence, ranging from approximately 18% to more than 90%. Similarly high rates have been observed in multiple CBCT-based cohorts [13,14,15]. This variability likely reflects differences in tooth morphology and population characteristics, as well as heterogeneity in imaging protocols (including voxel size), observer interpretation, and study methodology [16,17,18]. For example, a CBCT-based assessment in a Taiwanese population reported notable MB2 detection patterns, underscoring how cohort-specific anatomy and imaging/reading protocols can influence prevalence estimates [19]. These findings reinforce the complexity of maxillary molar morphology and highlight the importance of standardized CBCT methodologies in both anatomical studies and treatment planning.

Modern CBCT systems now make it possible to investigate how patient characteristics such as age and sex, along with technical factors such as voxel size, may affect the visualization of complex canal systems [20,21,22]. Such knowledge is essential for improving diagnostic accuracy, optimizing imaging protocols, and guiding future developments in computer-aided and artificial intelligence-based diagnostic systems [23,24]. Furthermore, assessing whether the presence of MB2 canals shows bilateral symmetry can offer clinicians predictive insights when evaluating contralateral teeth [25]. In addition, we evaluated canal detectability across predefined root/tooth levels (“tooth segments”), which are described in detail in the Methods section. Beyond prevalence, spatial relationships among canal orifices at the pulp chamber floor may assist clinicians in locating additional canals; accordingly, chamber-floor morphometric approaches to MB2 localization have direct clinical relevance during access cavity preparation. This concept is supported by CBCT morphometric studies quantifying MB2 geometric position and inter-orifice distances at the pulp chamber floor; for example, studies reporting geometric relationships among MB1, MB2, and palatal orifices highlight potential clinical implications during access cavity preparation [26].

Therefore, the primary aim of this CBCT-based study was to determine the prevalence of the second mesiobuccal canal (MB2) in maxillary first and second molars and to assess its relationship with the main mesiobuccal canal (MB1), including whether MB2 remains separate or merges with MB1. Secondary aims were to evaluate whether MB2 prevalence and configuration vary according to patient factors (age and sex) and CBCT technical parameters (voxel size), and to assess bilateral symmetry between contralateral teeth. This observational study will be reported in accordance with the STROBE checklist.

## 2. Materials and Methods

This retrospective cross-sectional study was conducted at the Division of Oral and Maxillofacial Radiology, Faculty of Dentistry, King Abdulaziz University, Jeddah, Saudi Arabia. The study was conducted in accordance with the principles of the Declaration of Helsinki (1975, revised in 2013). The research protocol was approved by the Research Ethics Committee (REC) at King Abdulaziz University of the Faculty of Dentistry (REC No. 045-03-23, approval Date: 06/03/2023). All cone-beam computed tomography (CBCT) scans were obtained from the digital imaging archive of the Oral Radiology Department at King Abdulaziz University Dental Hospital. The scans had been previously acquired for diagnostic purposes, and all data were fully anonymized before analysis. This manuscript was prepared in accordance with the STROBE (Strengthening the Reporting of Observational Studies in Epidemiology) checklist for cross-sectional studies.

A total of 146 CBCT scans acquired between March 2023 and May 2025 were retrospectively reviewed. All CBCT examinations performed during this period were consecutively screened using the institutional database, and all scans meeting the eligibility criteria were included. Scans were included if they involved patients aged 16–60 years and displayed at least one fully developed maxillary or mandibular first or second molar with clearly visible root morphology. Scans with metallic restorations, posts, orthodontic appliances, or other significant artifacts that could obscure canal visualization were excluded. Teeth with incomplete root formation or pathological conditions affecting root anatomy were also excluded.

Eligibility criteria: Only CBCT scans that included at least one maxillary first or second molar with complete root development and clearly visible root morphology were eligible. Teeth were included only when the crown and roots were sufficiently captured to permit assessment of the mesiobuccal root canal system. Scans were excluded if image quality was compromised by metallic restorations, posts, orthodontic appliances, or motion/beam-hardening artifacts that obscured canal visualization. Teeth with incomplete eruption/partially erupted status that limited standardized assessment of the coronal reference landmarks, and teeth with incomplete root formation, were excluded. Teeth with previous root canal treatment (identified by the presence of root filling material, intracanal posts, or clear signs of prior endodontic access/restorative intervention) were excluded to avoid bias from treatment-induced morphological changes and to reduce confounding related to canal calcification. Teeth with pathological lesions or conditions affecting root anatomy (e.g., severe resorption, fractures, or extensive pathology that altered morphology) were also excluded.

All images were acquired using an i-CAT Next Generation scanner (Imaging Sciences International Inc., Hatfield, PA, USA) operating at 120 kVp, 3–8 mA, and an exposure time of 8.9 s. The field of view (FOV) ranged from 8 × 8 cm to 13 × 16 cm according to clinical requirements. Because this was a retrospective study based on clinically acquired scans, acquisition parameters (including FOV and voxel size) were not standardized and reflect real-world imaging protocols. The voxel size ranged from 0.20 to 0.30 mm and was recorded for each scan a priori to evaluate its influence on canal detectability. Voxel size was therefore treated as a study variable and all analyses involving canal detection were stratified by voxel-size category and/or included voxel size as a covariate to reduce potential confounding. All datasets were exported in DICOM format for secondary analysis. Given the retrospective nature of the dataset, voxel size was treated as a technical study variable and incorporated into the statistical analysis to account for its influence on canal visualization.

Image evaluation was performed using Planmeca Romexis software (Planmeca Oy, Helsinki, Finland; available at https://www.planmeca.com/dental-software/planmeca-romexis/ accessed on 21 March 2026) in a dimly lit environment on a 27-inch diagnostic monitor. Two board-certified oral and maxillofacial radiologists, each with more than ten years of experience, independently assessed the scans using multiplanar reconstruction in axial, coronal, and sagittal planes, with the ability to adjust brightness/contrast and to scroll through thin slices to optimize visualization. A calibration session involving 20 non-study CBCT scans was conducted to standardize canal detection criteria. A canal was recorded as present only when a distinct radiolucent lumen could be confirmed in at least two orthogonal planes to minimize false-positive interpretation due to artifacts or partial-volume effects. Inter- and intra-observer reliability were tested using Cohen’s kappa and intraclass correlation coefficients (ICC), both exceeding 0.85. Discrepancies were resolved by consensus.

The MB2 canal was identified when a separate radiolucent lumen was visible in at least two orthogonal planes, either originating independently from the pulp chamber floor or merging with the MB1 canal in the middle or apical third. Operational definitions for additional canals were applied consistently across all scans, and a canal was recorded as present only when it could be confirmed in at least two orthogonal planes. The morphology of the mesiobuccal or mesial root canals was classified according to the Vertucci system [1], which describes eight types of canal configurations ranging from single to complex merging or dividing canals. The anatomical and morphological descriptions were supplemented by a systematic review of maxillary first molar root morphology [2] and by CBCT-based population studies [3,4,5], which have reported MB2 prevalence and detectability patterns across different populations. Tooth segmentation and reference levels. For standardized assessment, each evaluated molar was divided into predefined tooth segments corresponding to clinically meaningful root levels: coronal third, middle third, and apical third of the relevant root (mesiobuccal root in maxillary molars; mesial root in mandibular molars). The thirds were defined by dividing the linear distance from the canal orifice level (pulp chamber floor) to the radiographic apex into three equal parts along the long axis of the root using multiplanar reconstructions. All observations and measurements recorded “by segment” refer to these predefined thirds unless otherwise specified.

For each tooth, the mesiodistal (M–D) distance between the MB/MB2 or M/ML canals was measured at the coronal, middle, and apical thirds of the root, and the buccolingual (B–L) distance was measured at the chamber floor. Each third was defined by dividing the radiographic root length (from the cemento-enamel junction to the radiographic apex) into three equal parts along the long axis of the root using multiplanar reconstructions. Linear distances were measured as the shortest canal-to-canal distance between the centers of the visible canal lumens on the selected slice at each level. All measurements were made in millimeters using the digital ruler in Romexis software (accuracy 0.01 mm) and confirmed on two perpendicular planes. When bilateral data were available, the mean of right and left values was used to assess symmetry. Mesiodistal (M–D) distances were recorded as the shortest linear distance between the center points of the visible canal lumens (MB1–MB2 in maxillary molars; mesiobuccal–middle mesial or mesiobuccal–mesiolingual in mandibular molars, as applicable) at each root third. Buccolingual (B–L) distance at the chamber floor was measured on the axial slice where canal orifices were most clearly visualized. Measurements were performed using the built-in digital ruler tool in Romexis, and each measurement was confirmed on two perpendicular planes to minimize parallax and slice-selection errors.

All data were tabulated in Microsoft Excel and analyzed using IBM SPSS Statistics v22.0 (IBM Corp., Armonk, NY, USA). Descriptive statistics were expressed as means, standard deviations, and frequencies. The chi-square or Fisher’s exact test was applied to compare MB2 prevalence between genders, sides, and molar types. Continuous variables were analyzed using the independent *t*-test or Mann–Whitney U test, depending on data distribution. Spearman’s correlation coefficient (ρ) was used to evaluate associations between MB–MB2 distance, voxel size, and patient age. Bilateral symmetry of MB2 occurrence was analyzed using Cohen’s κ and McNemar’s test, and statistical significance was set at *p* < 0.05.

## 3. Results

A total of 146 cone-beam computed tomography (CBCT) scans were analyzed, comprising 92 maxillary and 54 mandibular molar regions. The mean age of the study population was 34.2 ± 10.6 years (range 16–60), including 62 males (42.5%) and 84 females (57.5%). Most scans were acquired at a voxel size of 0.30 mm (65.8%), followed by 0.20 mm (23.3%) and 0.25 mm (11.0%) (Table 1).

### 3.1. Prevalence of Accessory Canals

The mesiobuccal 2 (MB2) canal was identified in 64.4% of maxillary first molars and 29.6% of maxillary second molars, showing a significant difference between molar types (*p* < 0.001). In contrast, the middle-mesial canal in the mandibular arch was detected in 17.8% of first molars and 6.4% of second molars.

Sex did not significantly affect MB2 prevalence (male 59.7% vs. female 68.4%, *p* = 0.31) (Table 2).

**Figure 1:**
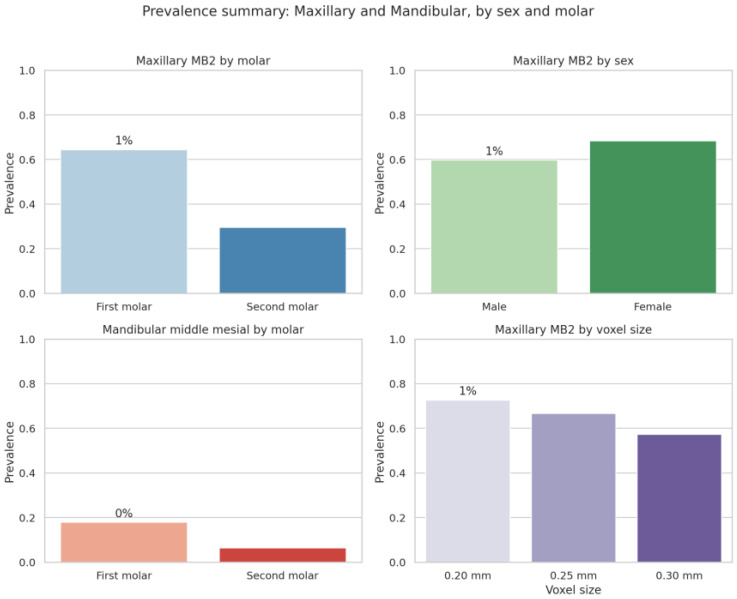
Prevalence summary: Maxillary and Mandibular, by sex and molar. Bar plots showing prevalence of MB2 and middle-mesial canals across molar types, sexes, and voxel sizes. MB2 detection was higher in first molars and at finer voxel resolution (0.20 mm). Appears after Table 2—summarizes MB2 and middle-mesial prevalence trends.

The prevalence patterns are summarized in Figure 1, which illustrates higher MB2 occurrence in first molars, a slight female predominance, and the strong impact of voxel resolution—detection declined from 72.7% at 0.20 mm to 57.3% at 0.30 mm. Middle-mesial canals were markedly less frequent overall, being more common in first than in second mandibular molars.

### 3.2. Morphometric Analysis

Linear morphometrics revealed progressive approximation of MB and MB2 canals from the coronal to apical thirds (2.01 ± 0.42 mm, 1.55 ± 0.38 mm, and 1.03 ± 0.33 mm, respectively).

The buccolingual chamber width averaged 1.86 ± 0.40 mm (Table 3).

Smaller inter-canal distances correlated with older age (ρ = −0.27, *p* = 0.02) and larger voxel size (ρ = −0.34, *p* = 0.01).

### 3.3. Mandibular Morphology

**Figure 2:**
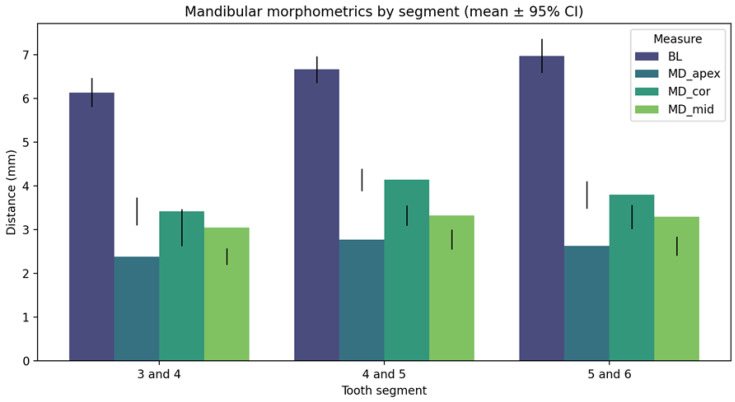
Mandibular morphometrics by segment (mean ± 95% CI). Distance measurements by tooth segment, showing the 4–5 region with the greatest inter-canal spacing and morphologic variation. Bars indicate the 95% confidence interval, and the black horizontal line within each bar indicates the mean value.

The mandibular analysis demonstrated measurable variation in canal spacing by tooth segment (Figure 2). The 4–5 segment exhibited the largest mean distances across all axes, indicating increased morphologic complexity in that region.

Middle-mesial canal detection varied by tooth, side, and sex but without significant asymmetry (*p* > 0.05) (Table 4).

### 3.4. Influence of Age and Voxel Size

A clear decreasing trend of MB2 detection with increasing age was observed—from 71.4% in patients <25 years to 53.6% in those ≥45 years (χ^2^ trend *p* = 0.020; Table 5). MB2 detectability also decreased with increasing voxel size, from 72.7% at 0.20 mm to 57.3% at 0.30 mm (*p*-for-trend = 0.018; Table 6).

Taken together, both increasing age and larger voxel size were associated with reduced MB2 detection.

**Table 5:** Age bands and MB2 detection (maxillary molars).

Age Band	*n*	MB2 Detected *n* (%)
<25 years	28	20 (71.4)
25–34	46	31 (67.4)
35–44	44	27 (61.4)
≥45 years	28	15 (53.6)

**Table 6:** Voxel-size distribution and MB2 detection (maxillary molars).

Voxel Size	*n*	MB2 Detected *n* (%)	*p*-for-Trend
0.20 mm	34	24 (72.7)	
0.25 mm	16	10 (66.7)	0.018
0.30 mm	96	55 (57.3)	

#### Bilateral Symmetry

Bilateral analysis revealed MB2 canals on both sides in 48.3%, unilaterally in 27.6%, and absent in 24.1% of cases.

Agreement between right and left sides was strong (κ = 0.78, *p* < 0.001).

For mandibular middle-mesial canals, bilateral presence was much lower (14.8%), with moderate agreement (κ = 0.29, *p* = 0.03). Check (Table 7) for details.

**Figure 3:**
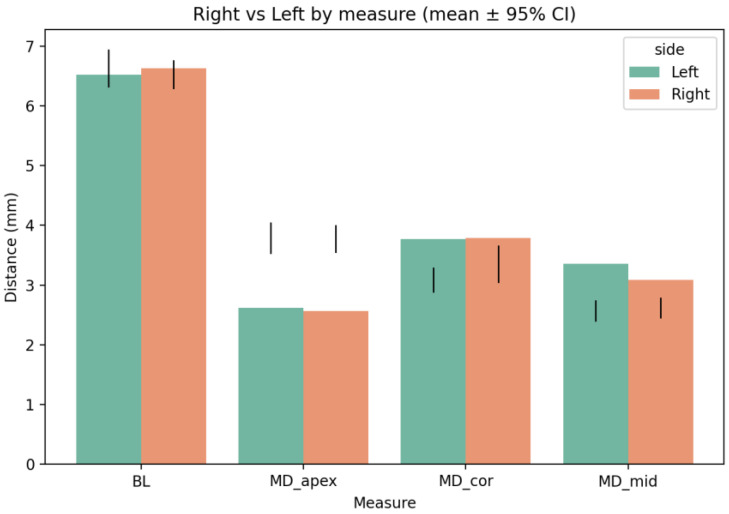
Right-versus-left comparison of mandibular morphometric measurements (mean ± 95% CI). Bilateral analysis shows minimal asymmetry between the right and left sides across all dimensional parameters. The central black line indicates the mean value, and the error bars represent the 95% confidence interval, demonstrating overall bilateral similarity in measurements.

Inter-side comparisons confirmed similar mean distances across all parameters (Figure 3), indicating high anatomic symmetry.

### 3.5. Vertucci Canal Configurationsfigure

Vertucci classification analysis (Table 8) showed Type I as the most common in maxillary MB roots (35.3%), followed by Type II (27.8%) and Type IV (22.1%).

In mandibular mesial roots, Type IV predominated (45.0%), followed by Type II (24.2%) and Type I (19.6%), reflecting higher canal interconnections in the mandibular molars.

## 4. Discussion

Understanding the intricate root canal system is essential for achieving predictable endodontic outcomes. This CBCT-based investigation evaluated the prevalence and morphology of the second mesiobuccal (MB2) canal in maxillary molars and the middle-mesial canal in mandibular molars, while also examining how patient- and imaging-related variables influence canal detectability.

In the present sample, MB2 canals were detected in 64.4% of maxillary first molars and 29.6% of maxillary second molars, reinforcing the established trend that MB2 anatomy is more frequently identified in first molars than in second molars [1,2,3,4]. For middle mesial canals, the apparent prevalence can be strongly influenced by the detection approach and whether troughing or targeted exploration is performed. Studies evaluating detection after guided troughing strategies suggest that additional canals may be uncovered when a systematic approach is applied, implying that “non-detection” in imaging-only studies does not necessarily equal true absence [23].

International comparisons showed similar patterns. Guo et al. found 66% MB2 detection in a North American population [16], whereas lower frequencies were reported in Iranian and Brazilian studies (42–52%) [14,15]. These between-study differences may reflect true anatomical variation, but they can also be amplified by differences in CBCT protocol and reporting quality. Contemporary recommendations in endodontics stress the importance of transparent and standardized reporting of diagnostic methods to improve comparability across populations and imaging systems [9].

When compared with earlier Saudi reports, our MB2 detection in maxillary first molars appears higher than values previously reported by Al-Fouzan et al. (53%) [19] and Al-Habib and Howait (58%) [20]. Alnowailaty et al. documented 56% detection using 0.30 mm voxels [21], while comparable Saudi CBCT studies have also reported variability in molar canal morphology [24]. This gap is plausibly explained by protocol-related factors, because spatial resolution and interpretation conditions can shift the likelihood of visualizing fine canal anatomy, especially when canal lumens are narrow or partially calcified. These observations align with the broader endodontic literature, which emphasizes that MB2 prevalence estimates are not purely anatomical but are also shaped by the diagnostic method and its reporting quality [9].

Voxel resolution was a key determinant of canal visualization in our analysis. Consistent with prior CBCT morphometric studies, chamber-floor spatial relationships among the MB1, MB2, and palatal orifices may provide practical localization cues during access preparation, underscoring the clinical value of three-dimensional preoperative assessment [26]. In our dataset, MB2 detection decreased from 72.7% at 0.20 mm to 57.3% at 0.30 mm (*p* < 0.05), supporting the view that clinically relevant anatomy may be missed when spatial resolution is reduced. Evidence-based reviews and technical guidance in endodontic CBCT consistently indicate that acquisition parameters and interpretation strategy influence diagnostic yield and should therefore be matched to the diagnostic task rather than applied uniformly across clinical indications [5,6,9,10]. Because this was a retrospective study based on clinically acquired CBCT scans, voxel size and field of view were not standardized; accordingly, MB2 prevalence and morphology estimates in the present dataset should be interpreted as potentially conservative, particularly in scans acquired at coarser voxel sizes. This limitation is consistent with the observed reduction in MB2 detectability with increasing voxel size

Patient-related factors, particularly age-associated calcification, can further reduce MB2 visibility even when CBCT is used. Prior investigations have reported associations between MB2 detection and patient age and/or the root third evaluated, suggesting that MB2 canals may be more readily identified in younger patients or when imaging conditions allow clearer visualization of fine anatomy in the coronal and middle thirds. These findings align with the clinical observation that progressive dentin deposition can narrow the MB2 lumen, making detection increasingly technique-sensitive and potentially contributing to inter-study differences in prevalence [11,12].

Because MB2 canals can be difficult to confirm on conventional imaging due to superimposition, comparisons between CBCT and periapical radiography are clinically relevant. Evidence comparing these modalities indicates that CBCT can improve identification of MB2 canals relative to periapical imaging, particularly when anatomy is complex or when additional canals are suspected but cannot be confirmed in two-dimensional views. This supports the rationale for considering CBCT in selected cases where the expected diagnostic gain is meaningful and consistent with appropriate-use principles [22].

Differences in reported MB2 prevalence are also influenced by the diagnostic modality itself. Comparative work evaluating CBCT against other approaches has shown that detection rates can vary substantially depending on whether the canal system is assessed by imaging, ex vivo clearing/staining methods, or other high-resolution techniques. This supports the interpretation that “true” anatomic prevalence and “observed” prevalence is not interchangeable, and that imaging-based estimates should be interpreted within the limits of the modality’s spatial resolution and susceptibility to artifacts [13].

At the same time, the range of MB2 prevalence reported across CBCT studies should be interpreted in light of methodological heterogeneity. Beyond voxel size alone, clinical interpretability can be influenced by reconstruction filters, contrast and sharpness settings, artifact burden from restorations, and differences in observer training and reading protocols. Therefore, prevalence estimates should be interpreted as a function of both anatomy and diagnostic conditions, and CBCT should be optimized to the diagnostic question while still respecting radiation protection principles and appropriate-use guidance [7,25].

Collectively, these findings support CBCT as a valuable adjunct for assessing complex root canal anatomy when conventional radiography and clinical exploration do not adequately explain symptoms, anatomy, or treatment difficulty. By reducing anatomic superimposition and enabling three-dimensional evaluation, CBCT can assist in understanding canal configuration and potential merging patterns, thereby supporting treatment planning when used appropriately [5,6,10]. Nevertheless, CBCT interpretation must be integrated with clinical findings and performed by appropriately trained clinicians to avoid over-interpretation of artifacts or incidental findings. As this was a retrospective study using clinically acquired CBCT scans, acquisition parameters were not standardized and may have contributed to under-detection of fine anatomy, particularly in scans acquired with coarser voxel sizes; therefore, MB2 prevalence and detectability estimates should be interpreted as potentially conservative.

Finally, future research should incorporate larger multicenter datasets, pre-specify acquisition and interpretation protocols, and report diagnostic accuracy outcomes using contemporary endodontic diagnostic-accuracy reporting guidance to strengthen comparability and transparency across studies [9]. Emerging automated and AI-assisted canal-detection approaches are promising, but will require robust external validation across scanners, protocols, and clinical settings before clinical translation into routine endodontic care.

## 5. Conclusions

This CBCT-based study provides comprehensive three-dimensional evidence of the prevalence and morphology of the second mesiobuccal and middle-mesial canals in maxillary and mandibular molars, respectively. MB2 canals were highly prevalent in maxillary first molars and strongly influenced by voxel size and patient age, emphasizing the diagnostic importance of fine-resolution CBCT imaging. The observed morphometric convergence and Vertucci configurations highlight the complexity and variability of molar canal systems. Bilateral symmetry supports the predictive value of contralateral anatomy during clinical assessment. Collectively, these findings reinforce CBCT as an indispensable tool for accurate detection and management of accessory canals, guiding clinicians toward more effective endodontic treatment and improved patient outcomes.

## Figures and Tables

**Table 1:** Demographic and acquisition characteristics of the CBCT sample (*n* = 146).

Variable	Category	*n* (%)	Mean ± SD
Age (years)	—	—	34.2 ± 10.6
Age bands	<25/25–34/35–44/≥45	28 (19.2)/46 (31.5)/44 (30.1)/28 (19.2)	—
Sex	Male/Female	62 (42.5)/84 (57.5)	—
Voxel size	0.20 mm/0.25 mm/0.30 mm	34 (23.3)/16 (11.0)/96 (65.8)	—
Arch coverage	Maxilla/Mandible	92 (63.0)/54 (37.0)	—

**Table 2:** MB2 (maxillary) and middle mesial (mandibular) detection by tooth and sex.

Group	Detection, *n*/*N* (%)	*p*-Value
Maxillary first molars	59/92 (64.4)	<0.001 vs. maxillary second
Maxillary second molars	27/92 (29.6)	—
Male vs. Female (maxillary MB2)	37/62 (59.7) vs. 58/84 (68.4)	0.31
Mandibular first molars (middle mesial)	10/54 (17.8)	—
Mandibular second molars (middle mesial)	3/54 (6.4)	—

**Table 3:** Linear morphometrics (mm) and correlations with age and voxel size (maxillary MB–MB2).

Measure	Mean ± SD	Range	ρ (Age), *p*	ρ (Voxel), *p*
M–D coronal	2.01 ± 0.42	1.1–2.8	−0.22, 0.04	−0.30, 0.02
M–D middle	1.55 ± 0.38	0.9–2.3	−0.27, 0.02	−0.34, 0.01
M–D apical	1.03 ± 0.33	0.5–1.8	−0.18, 0.08	−0.22, 0.05
B–L chamber	1.86 ± 0.40	1.0–2.7	−0.14, 0.12	−0.26, 0.03

**Table 4:** Mandibular mesial canals: middle mesial detection by tooth, side, and sex.

Variable	Category	*n*/*N* (%)	*p*-Value
Tooth	First molar	10/54 (17.8)	—
	Second molar	3/54 (6.4)	0.041
Side	Right/Left	7/54 (13.0)/6/54 (11.1)	0.74
Sex	Male/Female	6/62 (9.7)/7/84 (8.3)	0.78

**Table 7:** Bilateral symmetry of canal presence.

Arch/Canal	Both Sides	Unilateral	None	κ (*p*-Value)
Maxillary MB2	48.3%	27.6%	24.1%	0.78 (<0.001)
Mandibular middle mesial	14.8%	16.7%	68.5%	0.29 (0.030)

**Table 8:** Vertucci canal configurations by root.

Root	Type I	Type II	Type III	Type IV	V–VIII
Maxillary MB	35.3%	27.8%	12.8%	22.1%	2.0%
Mandibular mesial	19.6%	24.2%	8.9%	45.0%	2.3%

## Data Availability

The datasets generated and analyzed during the current study are available from the corresponding author upon reasonable request.
